# The value of a moment

**DOI:** 10.1017/S1478951525100874

**Published:** 2025-10-01

**Authors:** Alexander Hayes

**Affiliations:** 1 Department of Philosophy, King’s College London, London, UK; 2 NHS Greater Glasgow and Clyde, Glasgow, UK

After someone has died, I find myself wondering what they were like in life. If you’re lucky as a healthcare worker, you might have had the time to get to know them. We usually, though, only enter people’s stories at some of their most difficult chapters. By then, illness has often changed them, reshaping them from person to patient. That’s why I’ve always loved home visits. Even in illness, it’s easier for people to be themselves. They wear their own clothes, follow familiar routines, and surround themselves with photographs and mementos. Snapshots of both the big and the mundane, always in the company of their important people. If you’re particularly fortunate, you will also be offered endless cups of tea. Seeing patients in this way, I’m reminded of Susan Sontag’s idea that we are all born with dual citizenship: one in the kingdom of the well, and one in the kingdom of the sick. Where, “sooner or later each of us is obliged, at least for a spell, to identify ourselves as citizens of that other place.” (Sontag 1979) One way or another, illness comes for us all.

In medical school I wondered how best to help someone have a *good death* (Hayes 2022). Now as a Resident Doctor, it feels more experiential than definable. Even so, I think it helps to have some working idea of what it might look like. Some argue that the word *good* carries certain normative expectations. Namely, does a *bad* death mean something was *wrong*? *Ought* someone then be blamed? Sontag for instance, criticized the view that patients should be held morally responsible for their illnesses, arguing that sin is an erroneous cause of disease (Sontag 1978). Moreover, I think it’s worth noting that much of the language used in ethics is shared with art. Art may be *good*, but also beautiful, moving, unsettling, or even terrifying. A patient’s relative once described their loved one’s dying as, among other things, “interesting.” That curiosity stuck with me, it *was* interesting.

Starting work as a doctor I quickly realized that much of the job, at least at the beginning of one’s career, is about managing expectations and uncertainty, all the while often struggling to meet your own most basic needs. When I worked with acutely dying patients, in cardiac arrest teams and on the wards, I felt like I was witnessing repeated, faceless, deaths. I knew patients only by their deterioration. There were the: bleeders, can’t-breathers, heart stoppers. After resuscitation attempts, I noticed how many of us then, somewhat quickly, walked away, moving on to the next “task” on our lists. I started to feel a perpetual sense of guilt. Unsure why, I began lingering for a few minutes afterward. I would ask the nurses what they knew about the person. I would read their notes, searching desperately for a scrap of social history in their admission documentation. It felt as though any *goodness* in their death was beyond me, and all I could do was try to connect with this deceased citizen of the sick by learning their story. If not for them, then at least for myself.

There’s an adage in storytelling that good practice answers five key questions: who, what, where, when, and *why*. As best it could, I found my clinical education had prepared me for the first four. But on the rare occasion someone asked me a why question, I felt unprepared, confused and a bit scared. After all, why, can mean very different things to different people. The physicist Richard Feynman thought that the answer to a why question depends on the level of understanding of the person asking it. If for instance, you ask *why* someone slipped on the ice, the answer is simple. It is because ice is slippery. But if you ask *why* ice is slippery, that question he said, is “interesting.” The answer then would depend on how much you knew about the science of ice. Without some shared understanding, a why question cannot be answered in any terms that are familiar to the questioner (Feynman 1983). In medicine, why often feels more like an existential question than a scientific one. There is asking why in a causative sense, but that operates more as a *how*. Why did I get this cancer? Was it because of my smoking? But more importantly, *why* me?

At times, even asking, or answering a why in medicine feels impossible. When I really think about it, I am not even sure I can even conceptualize my own death. Freud thought no person could, stating that “It is indeed impossible to imagine our own death; and whenever we attempt to do so we can perceive that we are in fact still present as spectators.” (Freud 1957) In Damien Hirst’s *The Physical Impossibility of Death in the Mind of Someone Living*, a shark is enclosed and suspended in formaldehyde (Hirst 1991). Somehow, it appears, or is, simultaneously alive and dead. I wonder then what we can say when asked questions about death, if we don’t truly face it ourselves, except as spectators. If *pain is what the patient says it is,* then how do we best hear that? Is it enough to know that pain is painful, like how ice is slippery. How do we translate what we know to patients without reliance on metaphor, in a way that still allows space for “why”?

Our role is not to answer every question, but I think to create the space in which a question could be asked. I remember those 3am moments at work, staring into the pained eyes of another, that I find myself wondering what best to do for them. What do I do when at least one of us knows that death is coming. I recall John Berger, borrowing from Spinoza, who asks, “what is the value of the moment *sub specie aeternitiatis?”* (Berger and Mohr 2016 [1967]) From the perspective of the eternal, a moment is a very long time. I think I see a signal from them, and I wonder what it might mean, and what, if anything, I should do. Berger writes that *“*the anguished are trapped in a moment which is born of all that has happened to them” (Berger and Mohr 2016 [1967]). I don’t pretend to understand what they are feeling but I bear witness to this entrapment. After 43 hours on-call, I realize I cannot remember what I was doing an hour ago, or even ten minutes ago. From the perspective of the present, a moment is a very long time. I think I have an idea. I check the new medicine calculations, take a break, and check them again. I hope it will help. We both do our best. Caught between waiting and doing, with the sun beginning to rise, I head over to hand-over, bleary-eyed and barely orientated.

I remember T. S. Eliot,

“In the uncertain hour before the morning

Near the ending of interminable night

At the recurrent end of the unending” (Eliot 1942, II.3.1–3)

The moment passes. It is the end of the interminable night. They die, and I go on. I ask myself, what is the value of a moment? I hope I helped.

The nature of rotational medical jobs means you can go months without seeing someone die. Then, this person, a patient I spoke to every day, including about their approaching death, died. They said they were ready, but I don’t think I was ready. Cycling home from work, I kept thinking about the social dimensions of dying, about how the person still existed so long as I remember them, even after their death. I felt a sense of comfort that this somehow kept them alive. Joan Didion, in *The Year of Magical Thinking*, described the sense of unreality that followed the death of her husband: “There was a level on which I believed that what had happened remained reversible.” (Didion 2005). A colleague said that when patients ask why, it may be a way to seek reason in the face of insurmountable uncertainty. I remember hearing of one patient who had saved money for their holiday later that year, fully aware that they would likely die within weeks. When faced with the sting of death it somehow feels unreasonable to consider this unreasonable. The holding of seemingly mutually exclusive positions isn’t unlike us all knowing that one day we will die but being unable to really think about it.

The Abstract Expressionist Lee Krasner painted her *Umber Paintings* during an intense period of grief. These “Night Journeys” created whilst insomniac, were marked by a restricted, earthy palette and were a noticeable departure from her earlier work. It is hard to imagine how she must have felt while painting them, but I find them deeply moving. It feels like entering her story at one of its most difficult chapters. Color eventually returned to her work, and she painted for many more years. It seems then as if the dead remain with us, even as our story goes on. Krasner once said, “My painting is so autobiographical, if anyone can take the trouble to read it.” (Wagner 1996) I hope to take that kind of trouble, to learn the stories of those willing to share.
